# Empowering Shotgun Mass Spectrometry with 2DE: A HepG2 Study

**DOI:** 10.3390/ijms21113813

**Published:** 2020-05-27

**Authors:** Olga Kiseleva, Victor Zgoda, Stanislav Naryzhny, Ekaterina Poverennaya

**Affiliations:** 1 Institute of Biomedical Chemistry, Moscow 119121, Russia; victor.zgoda@gmail.com (V.Z.); snaryzhny@mail.ru (S.N.); k.poverennaya@gmail.com (E.P.); 2 Petersburg Nuclear Physics Institute named by B.P. Konstantinov of NRC “Kurchatov Institute”, Gatchina 188300, Russia

**Keywords:** proteome, transcriptome, proteoforms, alternative splicing, single amino acid polymorphisms, post-translational modifications, shotgun mass spectrometry, 2DE

## Abstract

One of the major goals of the Chromosome-Centric Human Proteome Project (C-HPP) is to catalog and annotate a myriad of heterogeneous proteoforms, produced by ca. 20 thousand genes. To achieve a detailed and personalized understanding into proteomes, we suggest using a customized RNA-seq library of potential proteoforms, which includes aberrant variants specific to certain biological samples. Two-dimensional electrophoresis coupled with high-performance liquid chromatography allowed us to downgrade the difficulty of biological mixing following shotgun mass spectrometry. To benchmark the proposed pipeline, we examined heterogeneity of the HepG2 hepatoblastoma cell line proteome. Data are available via ProteomeXchange with identifier PXD018450.

## 1. Introduction

Genes are the origin story about an organism’s development into what it finally becomes. Three billion nucleotide letters turn into bodies with their unique phenotypes and unique molecular portraits, which come out of the individual power of the genome.

Currently, a combination of postgenome technologies provides the opportunity to accelerate the understanding of molecular mechanisms of living systems. One of the most tantalizing postgenome objects to study are proteoforms [1]. Proteoforms are different protein products, encoded by one gene, which can differ dramatically from each other [2,3,4].

Alternative splicing (AS), single amino acid polymorphisms (SAP), and post-translational modifications (PTM) intrude upon the dominion of genes and crucially modulate biological processes due to the development of disease. Active attention to proteoforms made them a popular measure of postgenome objects. The deficit of information certainty about the heterogeneity of the proteome increases interest of this layer of biological information [5].

One of the most popular tools to obtain the fullest protein portrait of the sample under study is to interpret results of panoramic mass spectrometry (MS) through comparison of the experimental spectra and in silico spectra from search libraries. Relevant proteomic repositories usually provide data for search libraries containing not only canonical sequences but also aberrant variants. Unfortunately, even ready-made libraries for organisms with well-elaborated genomes do not allow for the performance of sample-specific research, because the reference library may differ from the proteome of the sample under study [6].

This study aimed to obtain the complete picture of the HepG2 cell line proteome, reflecting not only master proteins [7], which prove the expression of a certain gene, but also aberrant proteoforms, predicted at the mRNA level and comprising the heterogeneous proteome.

While investigating the HepG2 cell line, we followed the Chromosome-Centric Human Proteome Project (C-HPP) guidelines, which require two or more unique proteotypic peptides to be detected for confident identification of a proteoform. This requirement is quite challenging for analysis of aberrant proteoforms, because such proteoforms (especially inside one protein family) may have highly homologous or practically identical (in case of single amino acid changes) sequences. To obtain a personalized multiomics portrait of HepG2, we performed transcriptomic and proteomic analyses of the same cell line sample. In our study, two-dimensional electrophoresis (2DE) was not only a technique of complex protein mixture fractionation, but it was also a tool for proteomic analysis. Thus, 2DE provided the information about physical and chemical properties of proteoforms. This may be especially useful in terms of highly homologous proteoforms, which share proteotypic peptides but possess different molecular masses or charges.

Resulting data were used to profile the HepG2 proteome, to estimate the part of the proteome detectable by current analytical methods, and to speculate about the functional roles of certain proteoforms. Our research demonstrates that postgenomic methods based on sample-specific data allow for a more detailed view of the proteome.

## 2. Results

### 2.1. Customized Library: Size and Content

**Figure 1:**
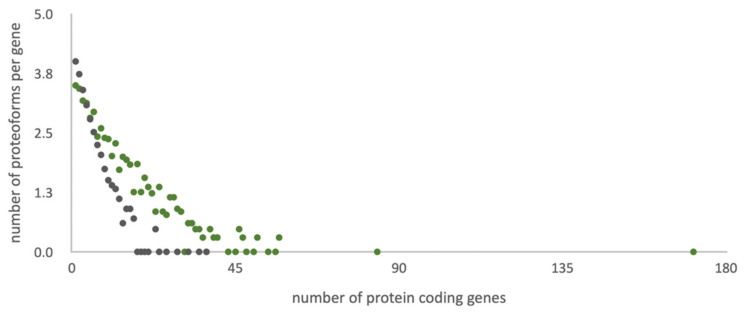
Distribution of the number of potential amino acid sequences per gene in different referent libraries. Grey dots correspond to standard UniProt library and green to the list of potential protein sequences of the HepG2 cell line (custom data).

Custom RNA-seq data for the biological sample of the HepG2 cell line were used as the basis for an in-house personalized library of amino acid sequences, which have the potential of realization at the protein level. This library contains 51,836 sequences encoded by 12,148 genes (~4.3 sequences per gene): 11,347 canonical sequences, 8489 splice variants, 8741 SAP, 9822 potential proteoforms with insertions or deletions, and 13,437 sequences with single amino acid changes, insertions, and deletions in alternatively spliced transcripts (Figure 1).

The UniProt library (42,424 sequences) is 20% more compact than the customized library considering that the standard library contains all human genes (20,408), and the RNA-tailored database contains only the entries (ca. half of the human genome) supported with evidence of transcription for the biological sample under study [8].

Figure 1 illustrates the character of the distribution of proteoforms per gene into two search libraries under study. The heterogeneity trend for the customized library is more gently-sloping than for the standard UniProt search list. Lowering of the trend angle is achieved by adding extra heterogeneity sources (e.g., point mutations, insertions, and deletions) and expelling the splice-variants with low chances of translation. Consideration of point mutations, insertions, deletions, and sample-specific splice-variants in the tailored library allows for equalizing search spaces.

According to the UniProt library, the maximum (37) of potential proteoforms falls on the *CAC1C* gene, which encodes the voltage-dependent calcium channel subunit Q13936. Interestingly, our transcriptomic studies of HepG2 did not reveal transcription of this gene, so potential sequences of Q13936 protein variants were not included in the customized reference library. In the custom transcriptomic data, obtained for the HepG2 cell line, the *PLEC* gene exhibits the maximum heterogeneity: There are more than 150 potential protein sequences for the corresponding giant protein plectin Q15149. A large amino acid sequence (4684 residues) is the platform for the realization of different combinations of nine splice variants, eight single amino acid polymorphisms, and nine insertions and deletions.

### 2.2. Comparison of the Libraries: Proteotranscriptomic Approach vs. Standard Search Space

**Figure 2:**
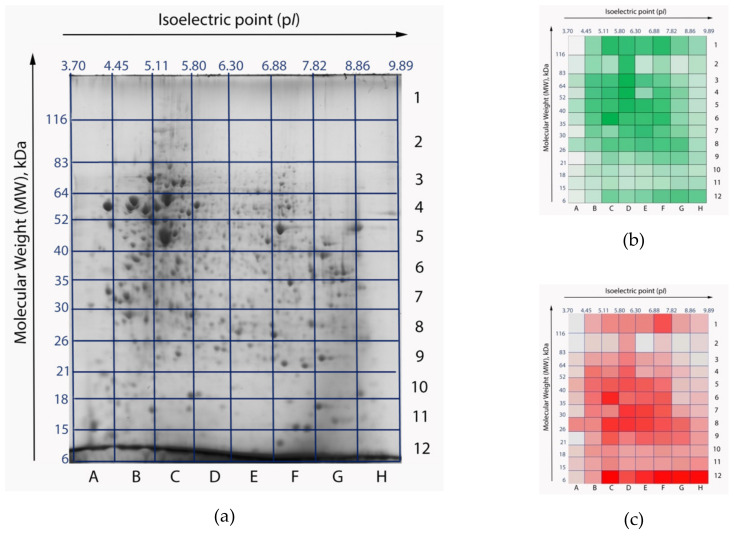
Two-dimensional electrophoresis (2DE) gel maps of the HepG2 cell line proteome: original stained gel with resolved proteins (**a**) [10]; heatmaps of peptide (**b**) and protein (**c**) identifications. Gel cells with a larger number of identifications are colored with high-intensity colors. The most intensive colors correspond to 4919 peptides and 193 proteins, respectively. Gel cells with a lower number of identifications appear pale. The least intensive colors correspond to 158 peptides and 5 proteins, respectively. Isoelectric points (p*I*) are marked as the *x-axis*, molecular weights (MW) are marked as *y-axis*.

To estimate the impact of the search library on the results of processing of the same experimental data, we searched the mass spectra corresponding to one of 96 gel cells (Figure 2), against two libraries: the custom RNA-seq-based library and the reference library downloaded from UniProt. We analyzed the E5 cell, which possesses one of the largest numbers of peptide identifications on this gel. This cell is located between p*I* 6.30–6.88 and molecular weights (MW) 40–52 kDa. Average values of isoelectric point and molecular weight of human proteins are ca. 7 and 61 kDa, respectively. Thus, protein molecules located in the E5 cell have average physical and chemical properties. 

When searching against the customized library, we detected 3599 and 3672 peptides in the first and second MS-replicates, respectively, with 89% overlap. Two technical runs resulted in forming a list of 3376 peptides, 663 of which were proteotypic (20%). Proteotypic peptides represent only one protein product in the proteome under study (Figure 2a). Among these findings were 35 proteotypic peptides that uniquely characterized aberrant proteoforms: 12 splice-variants, three SAP-variants, one combination of these two events, and five proteoforms with deletions. Other peptides (2713) were not exclusive, and mapped on several proteoforms encoded by one gene or by a group of related genes. We evaluated the number of unique protein groups. By protein groups we mean clusters of several protein products encoded by one gene or several related genes. Proteins inside a protein group share common peptides, which report on gene expression, but do not allow for reliable identification of a specific proteoform. In the cell under examination there were 14 single-gene protein groups and 500 protein groups encoded by several related genes. On average, each protein group consisted of six peptides. It is noteworthy that the number of unique protein groups allows for the estimation of a lower-bound of genome conversion comprehensiveness but does not provide reliable information on the proteome heterogeneity.

The search against the standard UniProt library resulted in 3519 peptides observed in the HepG2 cell line, 38% of which are proteotypic. It is expected that such a search would result in a lower proportion of identified unique proteins in comparison with the same search against the customized library. The reason for this is an extra way of performing particularization of aberrant events in the customized library, which downgrades peptide from proteoform-specific to gene-specific status.

The international standards of reliable protein identification require detection of a minimum two unique peptides [9]. Such a requirement is more than reasonable in the majority of proteomic studies, but the proteoforms encoded by one gene are often highly homological. This often makes the rule of two unique tryptic peptides unachievable. With that in mind, we weakened the restrictions of the identifications and accepted singleton proteins, which were revealed by one unique and one (or more) common peptides. Hence, in the cell under study, we identified 121 proteoforms encoded by 118 genes when searched against the tailored library (Figure 2c), and 217 proteoforms of 216 genes when searched against standard library. The search space of the customized library allowed for the detection of eight alternatively spliced variants, three proteoforms with point mutations, and one with a combination of these events. As a comparison, the usage of the UniProt reference library of human protein sequences resulted in identification of two alternatively spliced proteoforms.

**Figure 3:**
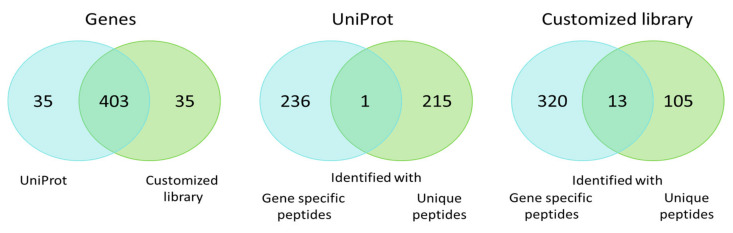
Venn diagram of genes for which proteoforms and protein groups were identified when searching against the standard UniProt library and the customized list of potential sequences.

Both libraries allowed for the detection of protein products encoded by 438 genes with 92% overlap (Figure 3). The protein products for one fourth of these genes were identified unambiguously to specific proteoforms. The protein products encoded by the remaining part of the genes were described as gene-specific protein groups when searching against the RNA-seq library. The UniProt library divided identifications into two groups: Protein products for 215 genes were identified as unique proteoforms, while protein products for the remaining 236 genes were identified as protein groups. On the one hand, addition of the heterogeneous transcript-specific sequence variants into the library raises confusion and uncertainty for the number of genes, reflecting the differences between their proteoforms. On the other hand, it gives the possibility of identifying the individual spliced variants and the proteoforms carrying SAPs, deletions, and insertions with increased reliability. The ambiguity of protein groups may be resolved with additional stages in data analysis, for example via exploration of the 2DE map, which allows for the specification of a certain proteoform in a protein group.

### 2.3. When Proteoforms Are Detected in Sudden Locations

We decided to use the customized library and searched through the MS reports corresponding to all 96 gel cells. The search against the customized library revealed 38,704 peptides, 8412 of which were proteotypic, mapping on sequences of 2156 proteoforms encoded by 2115 genes. In total, 1618 proteoforms encoded by 1589 genes were reliably identified. 

It is important to emphasize that only 33% of all identifications (530 proteoforms: 473 canonical ones, 27 splice-variants, 29 sequences with SAPs, and one case of amino acid change in an alternatively spliced variant) were found exclusively in a single gel cell. For each proteoform, we compared the theoretic coordinates of p*I* and MW calculated by the Pyteomics tool [11] with the coordinates of the gel cell where these proteoforms were found. Almost half (243 variants) demonstrated concordance in the theoretical and the observed localizations. For the rest, we analyzed the value and the direction of the experimental coordinates’ shifts in reference to the theoretical localization in order to determinate what caused these shifts.

One third of the one-cell group (163 proteoforms) was detected in the cells with lower values of molecular mass. Such migration to the lightweight zone is likely caused by proteolysis of a protein molecule into several fragments. Sequences of such fragments may contain proteotypic peptides required for reliable identification of a parent protein molecule; the molecular weight of the fragment is naturally lower than the weight of intact protein. The analysis of the peptides demonstrated that normally (71% of cases) peptides densely cover the whole protein sequence. Other shifts also took place, and they are discussed further.

### 2.4. When Protein Products of One Gene Are Found in Different Zones of Gel

#### 2.4.1. Post-Translation Modifications

We faced several cases when peptides mapping on the same proteoform were detected in remote gel cells. Besides the localization at the boundary of neighboring cells, simultaneous detection of peptides in different zones may be caused by a number of issues including PTMs, proteolysis, denaturation, isomerization, covalent binding of the protein molecule with its interactomic partner, etc.

According to the molecular mass (23 kDa) and the p*I* (5.98) of the heat-shock protein P04792, the identification of this protein is expected in the D9 or D8 cell, but the peptides of P04792 were also detected in eight other cells. The migration of P04792 to the area with lower molecular masses is likely associated with proteolysis (H12 cell, MW 6–15 kDa), and the simultaneous shift to the area with higher masses with a significant change of the p*I* (cells A7, B6, C2, MW 30–116 kDa) may be caused by homo- or hetero-oligomerization [12]. There is also a probability of artefactual C-C linking of P04792 with its interactomic partners: α- и β-tubulins, each of which weigh ca. 50 kDa [13]. 

**Figure 4:**
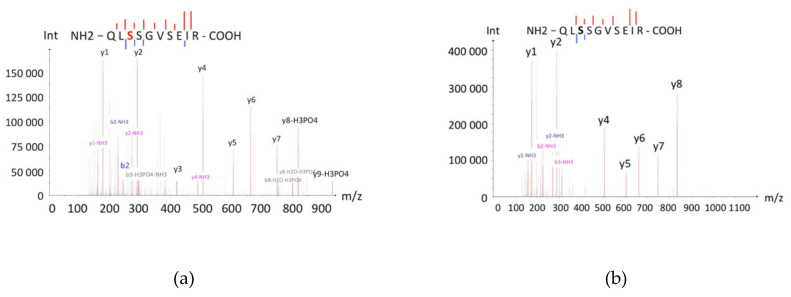
Mass spectra of the phosphorylated (**a**) and the non-phosphorylated (**b**) peptide QLSSGVSEIRs, which were detected in C8 and D8 cells, respectively. The series of peaks of the peptide indicate serine phosphorylation (S82). The non-phosphorylated peptide also has fragments, which indicates the absence of post-translational modifications (PTMs).

One of the most frequent post-translational modifications in terms of the isoelectric point value is phosphorylation, the substitution of neutral hydroxyl-groups of serine, threonine, and tyrosine with negatively charged phosphor-groups. As a consequence, the p*I* of the protein molecule decreases. This change can be significant; even occasional phosphorylation may decrease the p*I* of a protein by 1–2 points [14]. The shift of P04792 to the lower p*I* zone was supposedly caused by multiple phosphorylations of serine residues. According to the literature, the serine residues S15, S78, and S82 of P04792 are phosphorylated by MAP-kinases, which results in separation of P04792 from its oligomer, comprising several units [15]. In the C8 cell, corresponding to p*I* 5.11–5.80, we detected the phosphorylated peptide, containing the phosphorylated S82, while in the original D8 and D9 cells, PTMs were not observed (Figure 4). Apparently, only in two of 10 cells we could detect the real proteoform (the canonical sequence with minimal modification/phosphorylation), and the proteotypic proteins were detected in the other eight zones due to technical artefacts of sample preparation.

#### 2.4.2. Protein Complexes

We will consider the case of protein complex migration by the example of the oxidoreductase Q96HP4. Proteotypic peptides of this protein were found in cells F1 and F6. Supposedly, the F6 cell contained the canonical proteoform, with molecular weight (35 kDa) and charge (p*I* 8.86) corresponding to the cell parameters. The parameters of the F1 cell are compliant with less mobile proteins with larger molecular weight (>100 kDa). According to BioGRID, protein Q96HP4 interacts with seven partners, identified by affinity purification coupled with mass-spectrometry. The peptides, characteristic for the chaperone P10809 (one of the partners of the oxireductase Q96HP4) were detected in the same F1 cell. The molecular weight of this protein (ca. 61 kDa) is also inadequate for its autonomous migration to the F1 zone. Thus, it can be assumed that a covalent binding between the oxidoreductase Q96HP4 and the chaperone P10809 occurred during the sample preparation. 

**Figure 5:**
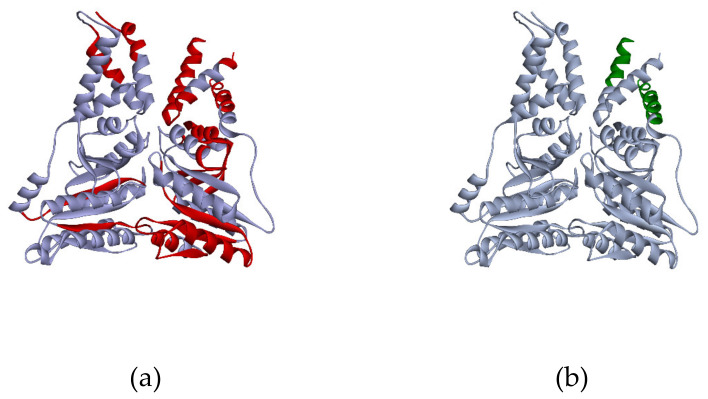
3D model of P61011 structure from PDB (Protein Data Bank). Red regions of the structure (**a**) correspond to the peptides, detected in the G4 cell, green regions (**b**) were found in the G10 cell.

Significant change of the amino acid content of the protein sequence after the proteolysis predictably results in the shift of protein localization to the lower weight zone. Moreover, such events may affect the p*I* of the truncated protein as could changes in the proportion between the positively and negatively charged residue infracts. According to our data, proteolysis was observed in ca. 25% of cases when the proteoform was identified in several gel cells. For example, the peptides of signal recognition protein P61011 were detected in two gel cells. Parameters of the G4 cell, which contained more than 20 peptides homogeneously covering the sequence (ca. 40% coverage), correspond with the theoretical MW of the protein. In the G10 cell, we detected only two peptides mapping the first α-helixes (Figure 5). It may be suggested that the proteins were broken into fragments in the biological sample or during the sample preparation.

Patterns of protein degradation provide more questions than answers. Thus, when finding the peptides of a protein in the cells in which MW parameters were lower than its theoretical molecular weight, we supposed that the protein was fractured into parts, and we did not consider such findings as individual proteoforms, e.g., splice variants.

**Figure 6:**
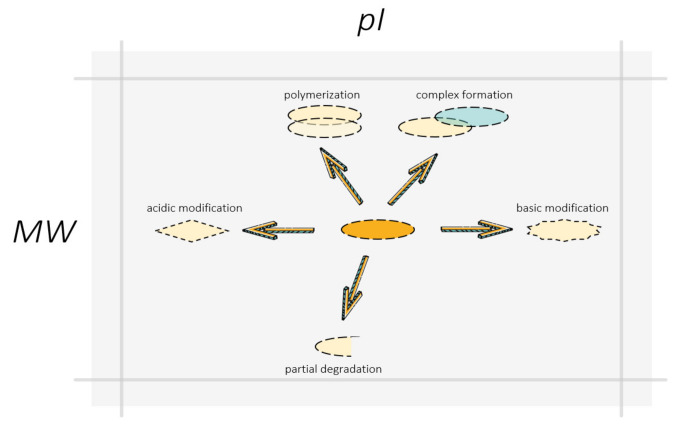
Swan, pike, and crawfish shifting proteoform localization on the gel: polymerization, complex formation, and non-complete denaturation moved proteins to a heavier zone, degradation reduced protein molecular mass, PTMs can change protein isoelectric points in both acidic and basic directions. The arrows indicate the directions of a possible shift in the localization of the proteoform on the gel resulted from different molecular events. A change in the shape of boxes symbolizes a change in the structure and molecular composition of the proteoform due to PTMs or degradation. The partner protein in the protein complex with the proteoform is represented by a blue box.

A significant portion of the identified proteoforms (1088 unique sequences, 67% of total identifications) were found in several (six on average) gel cells at the same time due to technical artefacts, proteolysis, or PTMs. To confirm the modifications, we re-analyzed the MS data concerning the most abundant PTMs: phosphorylation (serine, threonine, tyrosine), acetylation (lysine), methylation (lysine, arginine), amydation of C-terminal residues, and deamydation (asparagine, glutamine). Further analysis involved comparison of the physical and chemical parameters of the proteins and their possible modifications with the MW and the p*I* values of the cell, where specific peptides were detected (Figure 6).

### 2.5. Resolving Power of 2DE

The ambiguity of protein products annotation moves beyond the detection of peptides in non-typical zones of the gel. Another type of questionable identification consolidates the cases of protein group findings, which includes several protein products encoded by the same gene. In fact, 6950 protein groups were detected. At this point, with the support of the MS data only, it is impossible to determine certain proteoforms inside a protein group. Nevertheless, even these data are not a dead-end and could be successfully used for further analysis. Two-dimensional gel electrophoresis established itself as a leading choice of biochemical methods: 2DE applied to proteomics is an approach to inspect the proteome and is also a fractionation technique, which sets the difficulty of the specimen under study to the resolution capability of the next method of the analysis and allows decision making of translating proteoforms. Comparison of the theoretical and the experimental coordinates can be effectively used as an extra form of control. The discovery of the proteoform in a cell in which p*I* and MW dramatically differ from the theoretical values can suggest present PTMs, proteolysis, degradation, and other molecular events, if other proteoforms (alternatively spliced or mutated) are not supported with the transcriptomic prognosis. The localization of a proteoform on the gel depends on the physical and chemical properties of the protein molecule and the features of the certain experiment. 

The ability of 2DE to concretize the identifications can be illustrated by the example of seven tryptic peptides, which mapped at five alternatively spliced forms of the tioesterase (O00154, O00154-4, O00154-5, O00154-6, and O00154-7). The comparison of the theoretical MWs and p*I*s of these proteoforms with the cell parameters demonstrated that only one alternatively spliced form (O00154-7) has characteristics relevant to the cell coordinates, thus, we suppose, that the 7th spliced form was detected. 

Such a strategy is useful for closer definition of the alternatively spliced variants but not of the protein products with SAPs. In general, besides the polymorphisms, which critically affect the stability, the conformation, and the electrophoretic mobility [16], it is highly unlikely to concretize such a proteoform by means of its location in a large gel cell (ca. 0.7 points p*I* and 10 kDa of MW). 

### 2.6. Integrated Analysis of the 2DE Map

**Figure 7:**
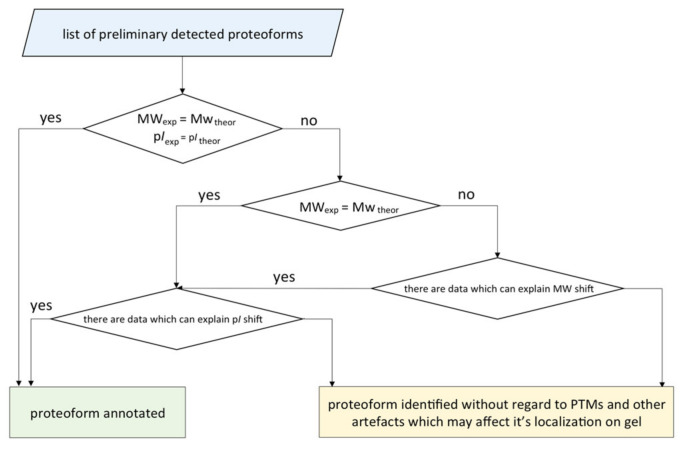
The scheme of the algorithm for annotation of the proteoforms detected by 2DE fractionation coupled with LC-MS/MS. A proteoform was considered as annotated if the proteotypic peptides were detected and the theoretical and the experimental coordinates of the localization were congruent.

We elaborated an algorithm for concretizing the proteoforms found in one or several gel cells (Figure 7). It started with listing the preliminary detected proteoforms and protein groups and then comparing the theoretical and the experimental coordinates of their localization. If the theoretic and the real p*I* and MW values matched, the proteoform was considered as identified. Otherwise, we investigated the reasons of the shift: multiple modifications, isomerization, proteolysis, degradation, and technical artefacts. Despite the consolidated efforts of biochemists and bioinformaticians towards the development of the algorithms for generating virtual 2DE maps, the task of predicting [17,18,19] the coordinates of localization is still far from perfect. Nevertheless, the tandem of panoramic MS and complementary 2DE looks promising in terms of distinguishing highly homological proteoforms. The values of p*I* and MW of a native protein are important characteristics, which allow us to put forward the hypothesis on the proteoform sequence and its PTM status.

The result is that out of 6649 protein groups containing the protein products encoded by a single gene, 751 individual proteoforms were identified. It should be noted that 10% of the protein groups contained sequences with SAPs, which can be distinguished with the proteotypic peptides with a polymorphic position. For 301 protein groups that consolidate the protein products encoded by several related genes, we distinguished 25 proteoforms.

Using this strategy in the HepG2 cell line we identified 2358 proteoforms encoded by 1904 genes. Among them there were 1658 canonical sequences, 224 splice variants, 115 canonical sequences with polymorphisms, and 31 alternatively spliced proteoforms with polymorphisms. For 990 proteoforms, we identified PTMs (Table 1). 

In total, we found proof of protein expression for 8512 of 12,148 genes from the tailored library (Supplementary 1). Were compared the results of the 2DE-LC-MS/MS proteome profiling of the HepG2 cell line with the panoramic MS profiling of the same cell line without preliminary 2DE-fractionation. It was shown that 2DE allows for an increase in the number of identifications by 76% for two reasons: the decreased complexity of the biological mixture under study and the additional information about the physical and chemical parameters of protein molecules, which allows for the analysis of the vector of the proteoform shift on the gel.

## 3. Discussion

The most popular search algorithms for the interpretation of proteomic MS data compare in silico generated mass spectra, which correspond to the theoretic peptides from the reference library, with the experimental mass spectra obtained from the real biological sample [20]. The subtle balance between the content and the size of the search library is crucial to consider all the sequences of possible proteoforms with minimal irrelevant noise for a manageable fraction of false identifications and reasonable search times.

In the first part of our study we evaluated the effect of the selected library on the number of the identified proteoforms. At first glance, the direct comparison of the numerical results of the search against the standard and the customized libraries (based on the transcriptomic data for this sample) supports usage of the UniProt database: the standard library provided 5% more peptide detections and 80% more identifications of individual proteoforms (with canonical forms prevailing, only three identifications were alternatively spliced).

Taking into account the differences between the standard and the tailored libraries, we decided to use the RNA-seq library as a base, providing a full and unbiased picture of the transcriptome for a thorough proteomic study of the sample under study. Our supposition of the rationality of the customized database usage was in keeping with other publications on the heterogeneous proteome. The importance of the search library for interpreting the proteomic MS-data may be illustrated with the example of a multi-omics analysis of 14 stock HeLa samples from 13 international laboratories that demonstrated pervasive copy number variations, ploidy changes of chromosomes, single nucleotide polymorphisms, quantitative differences in the transcripts, and proteoforms assemblies between different passages [21]. The customized libraries based on the RNA-seq data allow for identification of the unannotated genetic and splice variants while reducing the number of the comparisons (as well as false discoveries and the time of search, simultaneously) to only those transcripts that are unambiguously expressed in the tissue.

The alternative search strategy consists of the enrichment of the standard search space with the sequences carrying the aberrations from populational databases. Disturbingly, addition of the sequences of potential proteoforms to the standard library, which correspond to the non- and the low-expressed transcripts from different biospecimens, dramatically extends the search space and the portion of false identifications [22,23], while individual aberrations of the sample under study, such as point mutations, insertions, and deletions may disappear. 

For example, a well-known tumor suppressor TP53, of which multiple mutations were detected in various tumors [24], may serve as an illustration of the avalanche-like growth of the search space. According to the custom RNA-seq data, the gene TP53 encodes nine splice-variants of the P04637 protein (the canonical form and four splice-variants, which could be affected by the change of proline residue to arginine residue). The page of P04637 on UniProt.org contains about 1.5 thousand different variants, which include neutral polymorphisms, insertions and deletions, as well as disease-associated mutations and products of RNA-editing. The methodical limitations of modern proteomic methods do not allow for the estimation of the compatible frequency of such events. However, if we assume that point mutations of the amino acid sequence may occur both in the canonical and the alternatively spliced variants, the description of all possible proteoforms encoded by one gene would require by the most conservative estimate more than 15 thousand sequences. The application of such a strategy to the whole genome could be prodigal for the search space.

Using the method of separation of 2DE gel into parts proposed by Naryzhny et al. [10], we added two more coordinates to the proteomic analysis of the sample, which allows for more accurate identification. Based on the published data we formulated several hypotheses about the observed localization of the proteins on the gel. For example, lipidation affects localization of a proteoform in a similar way, the lipophilic groups attached to the protein molecule enhance its hydrophobic properties and, as a consequence, the migration of the lipidated protein in the gel could become easier. Hydrophobic proteins tend to speed up the migration through 2DE-gel and vice versa. This may be explained by the manner in which SDS binds to proteins. The hydrophobic proteins bind more SDS than do the hydrophilic proteins and move faster in 2DE-gel [25].

In contrast, the localization shift to the larger masses zones could result from the oligomerization of proteoforms or the covalent linkage with protein partners or contaminants as well as multiple glycosylation. Binding of proteoforms to other proteins, which result in the detection of specific peptides in non-typical zone of gel, is illustrated by the example of the migration of the prostate-specific antigen complex with peptide-inhibitor SPAAT to the larger masses zone [26]. Such a shift was observed for 68 proteoforms of the group under study. Except for the covalent binding and the oligomerization, such shifts may be caused by the characteristics of the structure and the amino acid content of the protein.

We believe that it is important to take into account the characteristic properties of the structure and the amino acid content of the protein molecule when integrating the MS and the 2DE data. The high content of proline residues, which set the angular form of the polypeptide chains, is believed to significantly reduce the mobility of a protein in the gel [27]. Tumor antigen P53 (P04637) can successfully illustrate this hypothesis. The molecular weight of the canonical and the longest form of P53 is 43.5 kDa, but this protein normally runs as a 53 kDa molecule on the SDS-PAGE. On average, the frequency of proline occurrence in the human protein sequence is ca. 6%. The sequence of P53 contains 11% proline residues. The proline richness potentially obstructs the protein migration in the gel, that is why proline-rich P53 is observed in 10 kDa heavier zones of the gel than in its actual molecular weight zone [28]. Presumably, the mismatch of the theoretical and the experimental coordinates of 18 proteoforms of the test groups is associated with high (>10%) occurrence of proline in the sequences. 

Remarkably, the non-complete denaturation of a protein could also result in abnormal localization on the gel. The regularities of the localization are still not conclusively established, but, according to certain publications, ca. 20% of *β*-sheets and *α*-helixes retain their structure even after the denaturation [29]. The whole class of the kinetically stable proteins is resistant to proteases and denaturating agents. The conformation of such proteins (including transthyretin and avidin) is blocked due to their low structural flexibility. As of the writing this manuscript, where are no specialized repositories that could assemble the non-structured data about the kinetically stable proteins, so the hypothesis about the effect of high kinetic stability on the gel localization of a protein may become a target for future studies.

Forty-one proteoforms from the test group shifted to the more acidic p*I* zone in comparison to the theoretical coordinates. Supposedly, such shifts could be explained by multiple PTMs, which slightly change the weight of the protein molecule but significantly affect its total charge [30]. This hypothesis was supported by the data from GPMdb and iPTMnet for 24 proteoforms. We also carried out re-analysis of the mass spectra with consideration to the most frequent and well-studied types of PTMs: serine, threonine, and tyrosine phosphorylation, lysine and N-terminal acetylation, as well as deamidation of asparagine and glutamine. The chemical modifications (methionine oxidation and cystein carbamidomethylation) that occur during the sample preparation were also taken into account. Such events were detected in 85% of peptides [31].

Deliberately narrow lists of the chemical and the post-translational modifications under study are associated with the exponential growth of the computational efforts when expanding the number of the monitoring modifications of the modified amino acid residues [32]. The assumption that there are only two PTMs per peptide (of more than 400 types) leads to a 160,000x increase of the search space, and, as a consequence, the growth of the false discovery rates (FDR). The MS detection of the modification normally easier, much more difficult tasks are for researchers to avoid the temptation to search against all existing PTMs in natural modifications and to interpret the obtained data correctly.

The shifts to the basic zone are more intricate: There were five proteoforms shifted to the basic p*I* zone. For example, the peptides of the acidic importin (O95373, p*I* 4.7) were detected in the H2 cell, which has atypical p*I* values (8.9–10.0). A sparse group of modifications may result in a basic shift. One of the most frequent basic PTMs is C-terminal amidation [33]. Lysine methylation also may affect the charge on the NH_2_ group and slightly increase the p*I* value [34]. We examined these modifications as a possible source of the mismatch between the experimental and the theoretical localizations. It turned out that two of five proteoforms found in the basic zone were poly-methylated and poly-amidated. The shifts of the other three proteoforms may be caused by non-complete purification, denaturation, or other technical artefacts [12,35].

Of course, it is impossible to take into account all the molecular events resulting in the vectorial addition of the several perpendicular shifts on the gel. Nevertheless, such considerations of the above-mentioned shifts in a manually curated mode allows for the reduction in false-positive identifications on the one hand and, on the other hand, to isolate proteoforms within the protein groups.

## 4. Materials and Methods 

### 4.1. Construction of Reference Libraries

We used the cells from the same HepG2 cellular culture for both transcriptomic and proteomic analyses to provide the maximum sample personalization. In our study, the transcriptomic analysis followed by the proteomic profiling was a source of a non-redundant personalized library of potential proteoforms. Optimization of the search library serves as an extra backup of the realization of certain DNA and RNA variants at the proteome level, as well as an extra inlet filter for population data, which characterize the whole population, but not the sample in all its uniqueness [22,36,37,38,39].

The customized library was built on the results of high-throughput mRNA sequencing of the HepG2 sample independently by Illumina Genome HiSeq 2000 and Applied Biosystems SOLiD 4 (experimental data are available in NCBI via IDs SRX395473 and SRX390071) in three technical runs, each with average read lengths of 43 bp and 163 bp for Illumina and SOLiD, respectively.

RNA-seq results were combined due to a previously demonstrated high correlation of gene expression levels between two platforms [40]. We trimmed the search space taking into account only the transcripts with the “reviewed” status in the UniProt database, FPKM (Fragments Per Kilobase Million) > 0.1, and the coefficients of variation of RPKM (Reads Per Kilobase Million) not exceeding 30% in two of three runs, minimum. PPLine package [41] was used for deleting low-quality reads, mapping filtered reads to the reference genome, assembling, and translating the transcriptomic data into the list of potential amino acid sequences. Subsequently, 51,789 amino acid sequences comprised the customized library. 

Besides the customized library, which was tailored specially for the biological sample of the HepG2 cell line, we used the UniProt library (release 2019_01) as a standard reference database of the human amino acid sequences, containing 39,182 canonical entries and 3147 splice variants.

We added the list of sequences of 116 protein contaminants from the cRAP database (ftp://ftp.thegpm.org/fasta/cRAP/crap.fasta) to each library to decrease the risk of false positives.

### 4.2. Handling of the 2DE Experiment

We fractionated approximately 10 mln cells, containing 2 mg of protein, of the HepG2 cell line of the same biological sample, which was used for RNA-seq analysis. 2DE was performed in accordance with the classical method of Klose and O’Farrell in denaturating conditions [42,43]. The details of sample preparation and 2DE analysis are described in detail in [10]. 

### 4.3. MS-Based Proteomics

The obtained 2DE gel was cut into 96 cells (~0.7 cm^2^ each) with previously determined coordinates of MW and p*I*. Each cell was cut, shredded, and treated by trypsin. After trypsinolysis, mixtures of peptides were extracted from the gel and dried in a vacuum centrifuge SpeedVac (Thermo Fisher Scientific, Waltham, MA, USA). Then, the dry mixtures were prepared for the LC-MS/MS analysis by first dissolving them in 5% (*v/v*) formic acid and then injecting them onto a trap column Zorbax 300SB-C18 (Agilent Technologies, Santa Clara, CA, USA) Agilent HPLC system 1100 Series (Agilent Technologies, Santa Clara, CA, USA). The peptides were fractionated on a 150 mm × 75 μm Zorbax 300SB-C18 reverse-phase analytical column (Agilent Technologies, Santa Clara, CA, USA) over a 30 min organic gradient of 5−60% (*v/v*) acetonitrile, 0.1% (*v/v*) formic acid, and a flow rate of 300 nL/min. The peptides were ionized by nanoelectrospray at 2.0 kV. Tandem MS was performed in two replicates on an Orbitrap Q-Exactive mass spectrometer (Thermo Fisher Scientific, Waltham, MA, USA). The mass spectra were acquired in positive ion mode.

The MS/MS spectra were extracted, converted into mgf format, analyzed using the combination of three search engines (X!Tandem (the Global Proteome Machine Organization, New York University Langone Medical Center, New York, NY, USA; the University of Manitoba, Winnipeg, MB, Canada), MS-GF+ (Pacific Northwest National Laboratory, https://omics.pnl.gov/software/ms-gf, Richland, WA, USA) and OMSSA (National Institutes of Health, Bethesda, MD, USA)), and implemented into the SearchGUI platform (v. 3.3.17, CompOmics, Ghent University, VIB, Ghent, Belgium) against the RNA-specific and the standard UniProt libraries. 

We used the following search parameters: enzyme specificity: trypsin; maximum one missed cleavage; carbamidomethylation of cysteine regarded as a fixed modification; oxidation of methionine, acetylation of lysine and N-terminus amino acid residues, and phosphorylation of serine, threonine, and tyrosine regarded as variable modifications; precursor mass tolerance of 10 ppm; and product mass tolerance of 0.01 Da. We ignored SAPs between I and L, which usually are hardly distinguishable by means of MS. To control the confidence of the identification, false discovery rates (FDR) were calculated in PeptideShaker by searching a decoy database. FDR < 1% was used as a cut-off criterion for all identified peptides and proteins. According to the international criteria of protein identification, the peptides with FDR > 1% and too short (<9 a.a.) or too long (>25 a.a.) peptides were removed from further processing.

The MS data were deposited to the ProteomeXchange Consortium via the PRIDE [44] partner repository with the dataset identifier PXD018450.

### 4.4. Proteoforms Identification

Two detected peptides (at least one of them should be proteotypic) were required for the protein identification. If the proteoforms, encoded by one gene, were supported by the shared peptides, which do not allow reliable distinguishing between the gene products, we claimed the identification of one protein group.

## 5. Conclusions

In accordance with the in-house transcriptomic data and the proteome size estimations, which suppose increases of PTMs and SAPs both in the canonical and the AS variants [4], the total number of proteoforms in the HepG2 cell line may vary between 124 and 306 thousand. The tandem use of 2DE and LC-MS/MS allowed for the detection of ca. 0.8–2% of this variety. The modest list of the found protein variants illustrates the difficulties faced by the scientists working in proteogenomics and proteoformics. There are a number of both biological and technical issues, which limit the chances of successful identification of a proteoform. Firstly, the combinatorial nature of reference libraries based on the transcriptomic data should be noted. The tailored library takes into consideration all possible variants of the amino acid sequences. Thus, if one of the RNA fragments contains the non-synonymous polymorphism, which maps onto the constitutive and alternatively spliced transcript, both variants will feature in the library. Secondly, not all transcripts are translated into proteins, for example, the stop-codon in an AS fragment will result in the degradation of the shorter transcript through nonsense-mediated decay—the mechanism preventing the cell from synthesizing potentially harmful truncated proteins. In addition, it is conceivable that there are retained introns that do not get out of nucleus and, as a consequence, do not translate. Thirdly, there are differences between the kinetic parameters of transcription and translation, when the transcripts are present at the RNA-level, but have not already translated into proteins at the time of RNA or protein isolation. Moreover, the proteoforms may not be detected due to their lower stability and abundance in comparison with the canonical variants. 

The identification of proteoforms via peptides could be hampered due to sequence redundancy/homology. Reliable detection of the shorter splice variants, which simply lack some parts of the sequence, becomes more complicated, because there is no specific peptide that could be used as a fingerprint for the absent fragment. 

The level of proteome coverage is not constant. The fraction of the identified proteofoms depends on the specificity and the sensitivity of the proteomic technologies. The progress of the analytical methods will naturally allow for the identification of more proteins. 

The body of proteomic data consolidates the new paradigm of system biology, which differs fundamentally from the classic dogma “one gene – one protein” [45]. In light of the new paradigm, the biological processes are presented as interactomic nets, which modulate the functions of each proteoform in the net. Thus, the next step in multiomics will be the transition from cataloguing of the protein molecules detected in different biological samples and conditions to identification of their functions through the protein maps specific for certain tissues, organs, and organisms.

## Figures and Tables

**Table 1:** The HepG2 proteome detected by panoramic MS in tandem with 2DE.

Type of Proteoform	Concurrent Theoretical and Experimental p*I* and MW		Chemical Modification		Proteolysis		Other		Total	
	P *	G **	P *	G **	P *	G **	P *	G **	P *	G **
Canonical form	481	481	57	57	107	107	107	107	752	732
Spliced variant	185	174	5	5	5	5	5	5	200	184
SAP form	42	39	6	6	6	6	5	5	59	55
SAPin spliced variant	24	23	2	2	1	1	0	0	27	25
PTM	527	527	124	124	163	163	92	92	906	858
PTM in spliced form	17	17	1	1	3	3	3	3	24	24
PTM in SAP form	33	31	6	6	8	8	9	8	56	50
Spliced form with SAP and PTM	2	1	0	0	1	1	1	1	4	3
Insertions and deletions	329	254	0	0	1	1	0	0	330	255

* P—number of proteoforms; ** G—number of corresponding protein-coding groups.

## Supplementary Materials

The following are available online at https://www.mdpi.com/1422-0067/21/11/3813/s1. Supplementary 1. Lists of the identified and the annotated proteoforms.
