# Supplementary material for: Empowering Shotgun Mass Spectrometry with 2DE: A HepG2 Study
Source: Int J Mol Sci. 2020 May 27;21(11):3813. doi: 10.3390/ijms21113813 (PMC7312985; doi:10.3390/ijms21113813)
Supplement: Supplementary file 1 [file ijms-21-03813-s001.pdf]

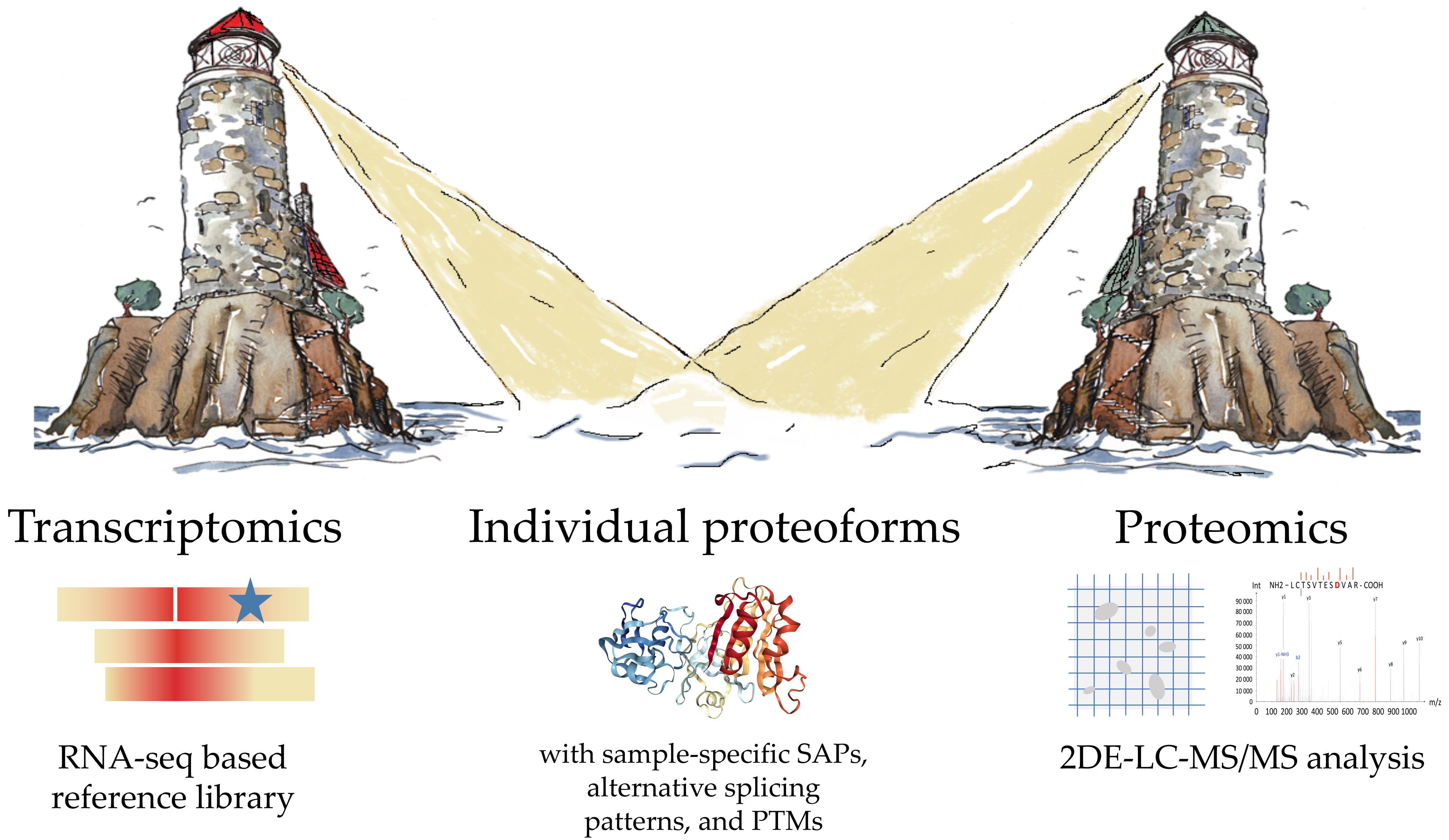
Transcriptomics
Individual proteoforms
Proteomics
RNA-seq based reference library
with sample-specific SAPs, alternative splicing patterns, and PTMs
2DE-LC-MS/MS analysis
Int
NH2 - LCTSVTESDVAR - COOH
m/z
